# A Notable Institution

**Published:** 1920-05-01

**Authors:** 


THE PUBLIC WELL-BEING—(*continued*).

### A Notable Institution.

Another instance has recently been brought to light of the miserably low scale of remuneration meted out to scientific research workers, whose services are of great value to the community as a whole. The Public Health Department of Manchester University, which was instituted in 1892 and was one of the first of its kind, is in need of funds, and the *Manchester Guardian* says that at present the stipends of the senior assistants, who have given over twenty years' invaluable service, range between £400 and £500 per annum. With the help of small grants it has been possible for the laboratory to meet the cost of its work, but this result has been achieved at the expense of the members of the staff, and has involved a restriction of the field of research.

A great deal of work is done on behalf of the Manchester, Salford, and Stockport Corporations; over 150 local authorities have also received its assistance, and its influence extends over an area including six counties, eleven county boroughs, seventeen municipal boroughs, sixty-six urban councils, and twelve rural councils, and investigations have also been made at times for various Government Departments. Original research work has been undertaken bearing on the causes and prevention of all classes of infectious disease, and its line of study has included the elimination of tuberculous milk from the market, the eradication of bovine tuberculosis, Asiatic cholera and plague, the study of frostbite, the protection of workers against noxious gases, pure water supplies, etc. It has also devised laboratory methods applicable to administrative work of investigating, controlling, and preventing diseases prevalent in the North-West of England, and the examination of morbid products has been a feature of the undertaking.

Such a service as this ought to be strongly backed financially and in every other possible way. First of all, the highly-trained scientific workers ought not to be expected to carry on such responsible tasks of wide utility at rates of pay which are scandalously inadequate.

